# A rapid assessment of migrant careworkers’ psychosocial status during Israel’s COVID-19 lockdown

**DOI:** 10.1186/s13584-020-00422-0

**Published:** 2020-11-02

**Authors:** Jordan Hannink Attal, Ido Lurie, Yehuda Neumark

**Affiliations:** 1 Braun School of Public Health and Community Medicine, Hebrew University of Jerusalem, Jerusalem, Israel; 2 Shalvata Mental Health Center, Hod HaSharon, Israel; 3 Department of Psychiatry, Sackler School of Medicine, Tel Aviv University, Tel Aviv, Israel

**Keywords:** Migrant workers, Caregivers, COVID-19, Mental health, Israel

## Abstract

**Background:**

Israel hosts nearly 70,000 migrant careworkers. Migrant careworkers work and live with populations extremely vulnerable to the novel Coronavirus, including the elderly and people with pre-existing physical conditions. This rapid assessment aimed to explore psychosocial status and mental wellbeing of migrant careworkers in Israel during the ongoing Covid-19 pandemic and determine risk and protective factors associated with mental distress, anxiety, and depression.

**Methods:**

This quantitative study was conducted via an online survey. The online survey collected social and demographic data, including country of origin, residence, age, sex, and time in Israel. In addition, questions were asked about knowledge of COVID-19 guidelines, access to supplies, and COVID-related racism. Respondents also completed a psychosocial screening tools, the Hopkins Symptom Checklist-10 (HSCL-10), which was used to screen for depression and anxiety.

**Results:**

As of May 3rd, 2020, 307 careworkers responded to the online survey, of whom 120 (39.1%) were found symptomatic using the HSCL-10. Separating the HSCL-10 into subscales, 28.0% were symptomatically anxious, and 38.1% were symptomatic for depression. In multivariate regression, emotional distress was associated with household food insecurity (OR: 5.85; *p* < 0.001), lack of confidence to care for oneself and employer during the pandemic (OR: 3.85; *p* < 0.001), poorer general health (OR: 2.98; *p* < 0.003), non-Philippine country of origin (OR: 2.83; *p* < 0.01), female sex (OR: 2.34; *p* < 0.04),, and inversely associated with age (*p* < 0.03). While 87.6% of careworkers reported having access to hand sanitization materials regularly, only 58.0% had regular access to a medical grade mask, and 21.5% reported household food insecurity. Moreover, 40.0% of careworkers claimed to lack confidence to care for themselves and their employer during the COVID-19 pandemic.

**Conclusions:**

Migrant careworkers exhibited high levels of mental distress during the COVID-19 lockdown, associated with lack of confidence or resources to properly care for themselves and their employer. Guidelines and support programs specific to the carework sector, that respect their rights and guard their health, must be developed as part of a coordinated COVID-19 response.

**Supplementary Information:**

The online version contains supplementary material available at 10.1186/s13584-020-00422-0.

## Background

Caring for the elderly and for persons with physical and mental disabilities or pre-existing medical conditions, migrant careworkers in Israel, and elsewhere, are the first-line defense in protecting those most vulnerable to infections and disease. This is especially true during the current the novel Coronavirus (COVID-19) epidemic when children and grandchildren were advised not to visit their elderly grandparents. The COVID-19 crisis added a considerable burden on careworkers who are responsible for their employer’s home and personal hygiene, running errands in the public sphere, and implementing national guidelines, while simultaneously safeguarding their own health, and worrying about the health of the family they have left ‘at home’. Furthermore, as the first COVID-19 cases were discovered in Israel in late February 2020, East Asian careworkers faced harassment and discrimination by Israeli citizens [1–3]. On dozens of videos that went viral in East Asian careworker social media groups, careworkers in public places were approached and told to leave the public sphere, or even the country because they likely have COVID-19.

At the end of 2019, Israel hosted nearly 70,000 migrant careworkers, most of whom were women and hail from Southeast Asian countries (See Table 1 for a detailed distribution by country) [4]. Migrant careworkers are live-in home health workers for Israelis who qualify for assistance and who meet legal and financial criteria set by the government. Most careworkers are employed by older adults, and others by persons with disability or parents of children with multiple disabilities [5].

**Table 1:** Number of migrant careworkers by country of origin according to PIBA, 2019 [4]

Country	N	%
Philippines	26,406	38.1
India	14,699	21.2
Moldova	9642	13.9
Uzbekistan	6303	9.1
Sri Lanka	4769	6.9
Nepal	2626	3.8
Ukraine	2410	3.5
Romania	670	1.0
Georgia	604	0.9
Other	1127	1.6
Total	69,256	100

Beginning March 19th, 2020, Israel declared a national state of emergency due to the COVID-19 pandemic, during which residents were instructed to remain in their homes except for accessing essentials- including purchasing supplies from supermarkets or the pharmacy or seeking medical attention [6]. On March 25th, 2020, more stringent restrictions were implemented by the government, most notably limiting residents to a 100-m radius surrounding their homes unless seeking essential services [6]. Further restrictions were added in the following 2 months, including city and neighborhood wide lockdowns and the requirement to wear facial masks in public [6].

Restrictions on leaving one’s place of residence led many manpower agencies, who manage the employment of migrant careworkers, to instruct migrant careworkers not to leave their employer’s home, and that under the situation, their legal right to a weekly day-off [3] was suspended [7]. Despite the Ministry of Labor and Social Services reinstating the right to a weekly rest-day as of May 7th, 2020, the Population and Immigration Authority published guidelines essentially forbidding careworkers from sleeping away from their employer on their day-off [8].

The conditions for acquiring a labor license to employ a migrant careworker include supplying adequate living space for the careworker. Nonetheless, many careworkers have been assigned to employers living in nursing homes and assisted-living facilities. These arrangements are illegal, though the laws prohibiting these arrangements are not well enforced. As COVID-19 hot spots developed in nursing facilities, careworkers became increasingly vulnerable to contracting COVID-19. The public interest in nursing facilities also led to the renewed interest of the Population and Immigration Authority (PIBA) and immigration police into the presence of migrant careworkers in nursing facilities.

Unlike many other high-income countries with migrant domestic workers, Israel does not allow a pathway to citizenship or permanent residency, and careworkers’ work visas are valid for a maximum of 63 months, with the opportunity to extend their stay in the country by obtaining a special visa [9]. In addition, in order not to promote permanent residency, careworkers are prohibited from having first-degree family members also working or living in Israel, including children. Children of careworkers born in Israel are returned to relatives in the careworker’s home country, or careworkers can make a request to both PIBA and their employer for their child to stay with them until the end of their visa period (up to 63 months), at which point both mother and child must leave Israel. It is important to note that such requests are rarely accepted by the authorities, and migrant careworkers who pursue this route are often threatened with the invalidation of their visa by PIBA, and lack of agreement of the employer puts the careworker’s job at significant risk [10, 11]. Violation of these terms leave careworkers liable to deportation, a vast wave of which occurred in the past year [12]. There are several laws governing careworkers, yet there is little monitoring as to the condition of careworkers themselves. Moreover, careworkers’ position in the private spaces of infirm, immobile, or aged Israelis, lends to invisibility among the Israeli public [13].

Careworkers are entitled to certain minimal conditions and services by law. These rights include a separate room in their employer’s dwelling, with a lockable door [9]. In addition, there are granted the right to a weekly vacation day and a daily two-hour rest period [9]. While careworkers with valid work visas are provided with health insurance, there are a number of limitations to services, including ambulatory mental health care [14].

Studies of migrant careworkers in many countries have indicated elevated mental health risk for this population even in non-pandemic circumstances [15–17]. In one Canadian study, for example, nearly a quarter (23%) of live-in careworkers surveyed indicated poor mental health and possible depression which were associated with long working hours and substandard living conditions [16]. Additionally, a 2019 study conducted in Israel found that emotional distress among Southeast Asian migrant careworkers was 36.8% using the HSCL-25 (Hannink 2020, unpublished thesis). Evidence from the current COVID-19 pandemic [18] as well as previous pandemics such as SARS [19] indicate increased incidence and prevalence of stress and anxiety disorders in the general public, amongst healthcare workers [20] and amongst migrant workers [21].

This rapid assessment survey of migrant careworkers in Israel has three major aims: 1) assess the psychosocial status and mental wellbeing of migrant careworkers during the ongoing COVID-19 pandemic; 2) determine risk-factors and protective-factors associated with mental distress, anxiety, and depression; 3) determine careworker knowledge of the outbreak and necessary precautions to prevent transmission. This study asked about stressors, outbreak knowledge, access to personal protective equipment, and experiences with COVID-related racism.

## Methods

Inclusion criteria for this online survey required that participants must be careworkers currently working in Israel, proficient in English, and have Internet access. Preliminary results were analyzed for careworkers who responded to the survey during the national lockdown, between March 31st and May 4th, 2020.

The questionnaire collected social and demographic data, including country of origin, visa area, residence arrangements, age, sex, and time in Israel. ‘Visa area’ refers to the geographic area a migrant careworker is assigned upon receiving a work visa, and is defined by PIBA as Area 1: Tel Aviv and its suburbs; Area 2: Jerusalem, Haifa and the Center, Area 3: the periphery [9]. In addition, questions were asked about knowledge of national COVID-19 guidelines, access to supplies, and COVID-related racism. In addition, careworkers were asked how confident they were to care for themselves and their employers during the COVID-19 pandemic, their general health status, and their sources of information for Israeli guideline updates and changes. Access to various resources and personal protective equipment was assessed, including access to handwashing and sanitizing materials, medical masks, disposable gloves, laundry facilities, their employer’s physician, and food for their employer and themselves. Some of these variables were coded into composite variables, including household food security, access to hand-sanitizing materials, and access to at least one type of medical-grade mask. Careworkers were also asked to report if they had personally experienced various events related to racism careworkers in Israel had experienced, including if they had been accused of having COVID, were threatened or harassed, had other people cover their nose/mouth while in their vicinity, had other people act as if they were afraid of them, or called names or insulted.

Respondents also completed the Hopkins Symptom Checklist-10 (HSCL-10), a psychosocial screening tool to screen for depression and anxiety [22] . The HSCL-10 is a short screening tool designed to identify common psychiatric symptoms in different cultural settings, and is considered an important and valid tool in transcultural research. The HSCL-10 is composed of two subscales: a 4-item anxiety scale and a 6-item depression scale. For each question, the respondent answers on a Likert scale: 1-“not at all,” 2-“a little,” 3-“quite a bit,” 4-“extremely”. The scores for the overall scale and the two subscales are calculated as the average of the frequencies of symptoms for items on each scale. Scores above 1.80 are considered above threshold and symptomatic for emotional distress (overall score), anxiety, and depression [22].

The survey was accessible online using a protected survey platform (SurveyMonkey, 2020), and distributed via careworker social media groups on Facebook and WhatsApp. This methodology was employed to enhance accessibility to this population which is known for its invisibility, though it is a sample of convenience. The online nature of the survey meant potential respondents could access the survey while on the job, and share the survey with their friends (snowball recruitment). We also were interested in maintaining methodology with our earlier (pre-COVID-19) survey of this population using the same online platform (Hannink 2020, unpublished thesis).

First-order statistics included χ^2^ and t-test to describe the socio-demographic characteristics of the respondents. A *p*-value of 0.05 or lower was considered significant. Multivariate regression modeling was used to measure associations between HSCL-10 scores and the socio-demographic variables, knowledge of COVID-19 guidelines, access to supplies, and the other variables addressed in the survey. A full list of variables included in the multivariate regression model is shown in Table 3. Analyses were performed using SPSS version 25 (Mac) (SPSS Inc., Chicago, IL).

## Results

As of May 4th, 2020, 307 careworkers responded to the online survey, of whom 78.2% were women (*n* = 226) or preferred not to say (*n* = 6), and 58.6% were from the Philippines (*n* = 180). Respondents ranged in age from 25 to 65 years (median = 37; IQR 32–43). Nearly half of the careworkers had been in Israel for more than 5 years. Complete socio-demographic information is presented in Table 2.

**Table 2:** Characteristics of migrant careworkers surveyed (*N* = 307) during COVID-19 pandemic, Israel, 2020

Variable		N	%
Sex	Male	67	21.8
	Female^a^	240	78.2
Age (years)	20–29	27	8.8
	30–39	154	50.2
	40–49	87	28.3
	50–59	25	8.1
	60+	1	0.3
	Missing	13	4.3
Country of Origin	The Philippines	180	58.6
	India	83	27.0
	Sri Lanka	28	9.1
	Nepal	3	1.0
	Romania	2	0.7
	Missing	11	3.6
Time in Israel	< 6 months	2	0.7
	6–12 months	13	4.2
	13–62 months	136	44.3
	> 63 months	152	49.5
	Missing	4	1.3
Visa Area^b^	Area 1	86	28.0
	Area 2	165	53.7
	Area 3	52	16.9
	Missing	4	1.3
Resident Status	Exclusively full-time with employer	154	50.2
	Lives with employer on work days; apartment for rest days	131	42.7
	Does not live with employer at all	19	6.2
	Missing	3	0.98
Health Status	Good/Very Good	248	80.8
	Average/Poor/Very Poor	43	14.0
	Missing	16	5.2

^a^included “prefer not to say” (*n* = 6)

^b^Area 1- Tel Aviv and its suburbs; Area 2- Jerusalem, Haifa, and the Center; Area 3- the periphery

One-hundred and twenty respondents (39.1%) scored above the overall HSCL-10 threshold, 28.0% were symptomatic on the anxiety subscale, and 38.1% were symptomatic on the depression subscale. Of the 115 who were symptomatic for emotional distress, 66 (55.0%) were found symptomatic on both subscales. On average, careworkers surveyed who scored above the HSCL-10 threshold reported 7.8 ± 1.7 symptoms, while those who scored below the threshold reported 3.1 ± 1.9 symptoms. There were no significant gendered differences in the average number of symptoms reported by those who scored above or below the HSCL-10 threshold.

In multivariate regression analysis, an above-threshold score on the overall HSCL-10 was associated with household food insecurity (OR: 5.85; *p* < 0.001; 95% CI: 2.64–12.94), lack of confidence to care for oneself and employer during the pandemic (OR: 3.85; *p* < 0.001; 95% CI: 1.88–7.89), non-Philippine country of origin (OR: 2.83; *p* = 0.01; 95% CI: 1.30–6.17), poorer general health (OR: 2.98; *p* = 0.03; 95% CI 1.09–8.11), female sex (OR: 2.34; *p* = 0.04; 1.28–6.11), and was inversely associated with age as a continuous variable (OR: 0.95; *p* = 0.03; 95% CI: 0.90–0.99). For the anxiety subscale, an above-threshold score was associated with household food insecurity (OR:3.00; *p* = 0.003; 95% CI 1.46–6.17) and poorer health status (OR: 3.60; *p* = 0.005; 95% CI: 1.46–8.88). For the depression subscale, an above-threshold score was associated with household food insecurity (OR: 3.64; *p* < 0.001; 95% CI: 1.77–7.52) and lack of confidence to care for oneself and employer during the pandemic (OR: 2.32; *p* = 0.013; 95% CI: 1.19–4.51). Results of multivariate regression analysis are summarized in Table 3.

**Table 3:** Multivariate analysis for HSCL-10 and its subscales among migrant careworkers during COVID-19 pandemic, Israel, 2020

		HSCL-10		Anxiety Subscale		Depression Subscale	
Variable		OR	95%CI	OR	95% CI	OR	95% CI
Age (years)	continuous	**0.95**	**0.90–0.99**	0.99	0.94–1.04	0.97	0.92–1.01
Sex	Female	**2.33**	**1.41–9.67**	1.56	0.77–4.84	1.89	0.84–8.17
	Male^a^	.	.	.	.	.	.
Region of Origin	Non-Philippine Origin	**2**.**83**	**1.30–6.17**	1.23	0.56–2.68	1.59	0.78–3.26
	Philippines	.	.	.	.	.	.
Time in Israel	< 6 months	1.00	0.03–29.12	2.63	0.12–57.20	1.45	0.06–36.00
	6–12 months	0.83	0.14–5.04	0.98	0.17–5.45	1.49	0.30–7.36
	> 63 months	1.70	0.86–3.34	1.26	0.64–2.50	1.34	0.71–2.53
	13–62 months	.	.	.	.	q	.
Visa Area^b^	Area 1	0.93	0.35–2.46	1.76	0.61–5.06	0.93	0.38–2.30
	Area 2	0.78	0.33–1.89	1.75	0.67–4.58	0.87	0.38–2.00
	Area 3	.	.	.	.	.	.
Residence	Lives with employer on work days; apartment for rest days	1.74	0.86–3.53	1.35	0.65–2.77	1.47	0.76–2.85
	Does not live with employer at all	3.06	0.60–15.49	2.83	0.70–11.37	4.85	0.99–23.67
	Exclusively full-time with employer	.	.	.	.	.	.
General Health	Average/Poor/Very Poor	**2.98**	**1.10–8.12**	**3.60**	**1.46–8.88**	2.24	0.89–5.59
	Very Good/Good	.	.	.	.	.	.
Employer’s Family Visits Frequency during Lockdown	More than usual	1.07	0.38–3.00	0.79	0.28–2.23	1.16	0.44–3.07
	Less than usual	1.12	0.50–2.52	0.73	0.32–1.64	0.91	0.43–1.96
	about the same as usual	.	.	.	.	.	.
Access to hand sanitizers	Lower access	0.96	0.33–2.85	1.39	0.47–4.15	1.11	0.40–3.07
	Higher access	.	.	.	.	.	.
Personal Protective Equipment	Low availability	1.40	0.55–3.61	0.70	0.27–1.85	1.75	0.71–4.32
	Moderate availability	1.04	0.47–2.30	0.72	0.33–1.59	1.30	0.61–2.77
	High availability	.	.	.	.	.	.
Household Food Security	food insecurity	**5.85**	**2.64–12.94**	**3.00**	**1.46–6.17**	**3.64**	**1.77–7.52**
	Adequate food security	.	.	.	.	.	.
Laundry Access	Never	0.38	0.08–1.80	0.23	0.03–1.76	0.40	0.08–1.79
	Sometimes	0.34	0.11–1.10	0.36	0.10–1.33	0.41	0.13–1.26
	Always	.	.	.	.	.	.
Confidence to Care	Not Confident	**3.85**	**1.88–7.89**	1.80	0.90–3.62	**2.32**	**1.19–4.51**
	Confident	.	.	.	.	.	.
COVID-19 Related Racism	Experienced racism	1.38	0.72–2.62	0.96	0.57–2.25	1.42	0.78–2.61
	Did not experience racism	.	.	.	.	.	.

^a^included “prefer not to say” (*n* = 6)

^b^Area 1- Tel Aviv and its suburbs; Area 2- Jerusalem, Haifa, and the Center; Area 3- the periphery

### Access to personal protective equipment

Among careworkers surveyed, 87.6% (*n* = 269) had ample access to hand-sanitizing materials, and 58.0% (*n* = 178) always had access to some type of medical-grade mask (Table 4). Despite being their employer’s primary caregiver, 44.0% (*n* = 135) did not have any contact with their employer’s primary care physician. When considering both the employer’s and caregiver’s access to food, 21.5% (*n* = 66) careworkers lived in homes with food insecurity. There were no differences in access to supplies based on the length of time careworkers had been in Israel.

**Table 4:** Access to resources and personal protective equipment among migrant careworkers during COVID-19 pandemic, Israel, 2020

Variable	Access		
	Always (%)	Sometimes (%)	Never (%)
Access to Hand sanitizers			
Access to Soap and water	96.6	1.4	2.1
Access to hand sanitizer	68.1	23.4	8.5
≥ 1 hand sanitizing method	98.7	1.4	
Facial Masks			
Surgical masks	55.5	34.1	10.3
N95 masks	18.7	14.8	66.6
≥ 1 type of medical-grade mask	57.9	42.1	
Other Resources and PPE			
Disposable Gloves	68.7	26.8	4.5
Laundry Facilities	87.5	7.5	5.1
Contact with Employer’s Doctor	15.7	38.3	46
Food Security			
Food for Employer	87.9	9.3	2.8
Food for Self	84.5	14.1	1.4
Overall Food Security	78.5	21.5	

### Access to COVID-19 information

Careworkers reported receiving COVID-19 information and guideline updates primarily from English-language Israeli news sites (*n* = 268) and from non-government organizations in the careworker sector (e.g., Kav L’Oved and HaKeren) (*n* = 124). On average, careworkers received updates on COVID-19 from 2.27 (±1.45) sources.

### Experiences of COVID-19 racism

More than half (55.0%; *n* = 169) of the respondents reported having experienced at least one instance of COVID-19 related racism. As seen in Table 5, the most frequently reported experiences included having people cover their faces when walking past the careworker (43.0%). Among the 53 careworkers who responded to the survey before masks were mandatory when in public spaces, 37.0% claimed that people had covered their faces when walking past the careworker and 39.1% felt that people acted as if they are afraid of them. Notably, 25.7% of careworkers reported that they were accused in public spaces of having COVID-19, with no just cause.

**Table 5:** COVID-19 related racism experiences among migrant careworkers during COVID-19 pandemic, Israel 2020

Experience	n	%
People covered their faces walking past you	132	43.0
People act as if they are afraid of you	120	39.1
Called names or insulted	86	28.0
Accused of having Corona	79	25.7
Threatened or harassed	36	11.7

## Discussion

The prevalence of anxiety, and depression observed in this study among migrant careworkers in Israel is remarkably high in comparison to the prevalence of these mental health conditions in the careworkers’ countries of origin and in the general population of Israel [23], and similar to that among Asian migrant communities in high income countries [24–27] (Hannink 2020, unpublished thesis). A meta-analysis of anxiety and depression screening studies among Asian immigrants in the US reported depression prevalence rates in the range of 3 to 71% using the HSCL and other similar screening tools, with the highest rate noted among Korean American family caregivers [18, 19]. The pooled estimates from this meta-analysis were 27–36% [18]. These studies were not conducted during a global pandemic, but provide a baseline for analyzing the prevalence observed during the COVID-19 pandemic.

The present study found a two-fold greater likelihood (OR = 2.3) of an above threshold HSCL-10 overall score among women. Gendered differences in the rates of anxiety and depression are documented across cultures, though the mechanisms for these differences remain unclear [28–31]. In light of the gender differences, some suggest that the thresholds used for mental health screening tools should be different for men and women. Some studies suggest that the threshold for women should be higher, while for men the threshold should be lower [28, 29].

This association may be rooted in gendered differences in psychiatric symptoms resulting from sexist treatment in everyday life. In one study, women who reported experiencing frequent sexism also exhibited a greater number of depressive and somatic symptoms then men, whereas women who reported experiencing little sexism exhibited similar numbers of symptoms as men [30]. Sexist discrimination may account for nearly half of the variance in depressive symptoms [30, 31]. In addition to blatant sexism, gendered expectations may influence one’s sense of duty to care. These existing gendered expectations to care may have direct consequences during pandemics. Historical data from the 1918 Spanish Flu pandemic in Canada have shown that women were placed at greater personal risk due to a sense of moral obligation to care [32]. In the present study, the association between female sex and a score above the threshold on the HSCL-10 may relate to a gendered understanding of obligation to care [33].

Similar to the findings presented above, mental health outcomes have been linked to self-reported general health in several studies, especially among Asian populations [34, 35]. This may be in part due to a strong conceptualization of the mind-body connection, which results in more somatic symptoms [36, 37]. Pandemic conditions may intensify the effect of the mind-body connection for those who already feel that they are not healthy, and therefore possibly more vulnerable to infection [38].

Confidence, or lack thereof, has been bi-directionally associated with anxiety in a variety of situations [39–41]. In a recently published review about anxiety among health care professionals during the COVID-19 pandemic, sources of anxiety included access to proper personal protective equipment, being exposed to COVID-19 and taking it home to one’s family, uncertainty that their place of employment will take care of their needs if they become ill, and the demands of additional hours at work [42]. Additionally, lack of confidence may be attributed to language barriers [43]. Information about the COVID-19 situation and translations of national guidelines, including in Tagalog (Wikang) and Hindi, were published by the MoH and distributed through governmental and select non-governmental organizations. These guidelines were difficult to find online, especially if one lacked ability to read Hebrew. Some languages, including Hindi and Tagalog, were not accessible on cell phones.

Lacking access to personal protective equipment was an alarming finding in this study, particularly that 42% of careworkers claimed not to have access to facial masks. This finding was considered relatively high, considering masks were required to enter public spaces during data collection and their use in the home is in the best interest of the employer and the careworker. The high proportion of careworkers who reported not having access to face masks may relate to unresolved questions as to who is financial responsible for face masks- the careworker as a personal expense, or the employer and/or their family as a business expense. For careworkers who were instructed to not leave the home of their employer during the lockdown, careworkers may not have had the opportunity to purchase masks, and rely on their employer’s family for maintaining supplies. However, lack of access to personal protective equipment was not found to be associated with an above-threshold score on the anxiety or overall HSCL-10 scales. Further, there were no observed differences in access to supplies between careworkers who had been in Israel for longer periods and shorter periods, suggesting that accessing supplies is multidimensional, rather than a simple function of experienced-acquired knowledge.

Perceived household food insecurity was consistently significantly associated with an above-threshold score on the HSCL-10 overall scale and the anxiety and depression subscales. In a study conducted by the Israel Central Bureau of Statistics during the coronavirus lockdown among a representative sample of the Israeli population, 14% of respondents reported that they or members of their household reduced their food intake or meals they had eaten in the week prior to the survey due to economic insecurity [44]. In the present study, 21.5% of respondents reported household food insecurity, which may indicate the increased vulnerability to food insecurity during the COVID pandemic among the elderly, disabled, and minimally paid careworkers. In a global analysis of 149 countries, food insecurity was associated with poorer mental health and specific psychosocial stressors, regardless of socioeconomic status [45]. Other studies have explored the link between food security, mental distress, and sex [46, 47]. In one study, women who reported food insecurity were twice as likely to report psychological distress as men who reported food insecurity [48]. Sex did not modify the relationship between household food insecurity and five adverse mental health outcomes in a study of Canadian adults [49]. For careworkers, mandatory confinement during and after the COVID-19 lockdown may have limited their pathways to food for themselves and/or their employer. While online grocery services and delivery are available, navigating the websites and entering the order requires knowledge of written Hebrew, which many careworkers do not possess. The absence of the employer’s family may have further contributed to disrupted supply pathways. In anticipation of possible lockdowns in the future, plans to ensure food security for the elderly and their careworkers, must be put into effect.

### Study strengths and limitations

The online nature of the study allowed easy access to the survey, and eliminated geographic and temporal limitations. In order to ensure the acceptability of the survey among careworkers, the survey was shared in social media groups of institutions that careworkers trusted (KLO), and thus trusted the source of the questionnaire. In other social media groups for careworkers, careworkers who had personally chatted with the researcher would often vouch for the study and the researcher, often in the group’s mother tongue, which made the survey acceptable among careworkers in those spaces. However, the results of this study must be viewed in light of several possible limitations. Firstly, this study relied on the HSCL-10 to establish prevalence of symptoms of mental distress. While this screening instrument is widely used and has been shown to be valid in diverse settings [50, 51], it is not equivalent to a clinical diagnostic evaluation. Screening tests are designed to identify persons who are likely to have the condition being screening for, who should then be referred for diagnostic confirmation and appropriate treatment when necessary.

The questionnaire addressed multiple aspects of careworkers’ lives that may influence their wellbeing, although the vulnerability of this population prevented certain questions from being asked, such as possession of a valid work visa. Not having a visa may have a significant impact on the careworker’s mental health status, particularly during the lockdown period when police presence was amplified throughout the country. However, asking about this, or other sensitive topics, would have caused heightened distrust among the potential respondent likely leading to non-response or false reporting. For this reason, the questionnaire did not ask careworkers if they had valid medical insurance, or if they were employed by a manpower agency or directly by the employer/employer’s family. A qualitative study with direct face-to-face interviews, may have allowed us to address these questions, however this was not possible during the lockdown.

In addition, the online nature of the survey leaves the study without a well-defined sample frame. In an attempt to be as accessible as possible- particularly during a national lockdown- by placing the survey online, careworkers without internet access or who choose not to be in social media groups for migrants and careworkers in Israel would not have had access to this survey. Simultaneously, careworkers who left Israel but remained in these social media groups maintained access to the survey, despite not meeting inclusion criteria. The researchers attempted to limit this potential sampling bias by clearly stating in the recruitment post that only careworkers currently working in Israel were eligible. Additionally, there is a growing cohort of migrant careworkers from Former Soviet Union countries. The survey’s availability only in English may have excluded many of these careworkers from participating. Lastly, the percentages of respondents from each country of origin do not reflect the composition of the migrant careworker community, and thus may limit the generalizability of results.

### Recommendations

In a recent publication concerning mental health during the COVID-19 pandemic [52], the WHO encourages healthcare workers to use helpful coping strategies, including sufficient rest and respite during work or between shifts, maintaining a healthy and adequate diet, engaging in physical activity, and staying in contact with family and friends. These guidelines and recommendations equally apply to careworkers, as essential workers in the health sector. Under current government guidelines, careworkers cannot practice adequate self-care. During this study, careworkers were prohibited from leaving the homes of their employers, and despite recently published guidelines for rest days by the Ministry of Labor, Social Affairs, and Social Services, the restrictions make it nearly impossible for migrant careworkers to take their rest day outside of their employer’s residence [53]. In addition, despite these regulations, careworkers working in assisted living facilities have been forbidden by the assisted living facilities themselves from leaving the premise [54].

Migrant careworkers are essential workers at the frontlines of defending the most vulnerable from a potentially deadly illness. In the next phases of COVID-19 pandemic response, and in preparation for future epidemic events, specific guidelines for migrant careworkers must be formulated by the Ministry of Health and Ministry of Labor, Social Affairs, and Social Services to protect the physical and mental wellbeing of both employer and careworker. These guidelines must be professionally translated into the national languages of careworkers’ countries of origin, as some careworkers do not have an adequate proficiency in Hebrew or English to clearly understand formal and detailed texts. In such situations, they often rely on informal translations by fellow careworkers, which may not be precise, potentially leading to misunderstandings and subsequent mistakes in complying with guidelines and properly protecting careworkers and their employers.

The Ministry of Health attempted to expand services through a government tender for the provision of mental health services for non-Israeli citizens through the Ruth Mental Health Clinic in Tel Aviv. The capacity of this clinic to absorb new patients turned out to be extremely limited, and was unable to meet the needs of migrant workers, asylum-seekers and undocumented migrants residing in Israel. Expressly in extraordinary circumstances, such as a pandemic-induced lockdown, it is the responsibility of the government and manpower agencies to ensure the physical and mental wellbeing of migrant careworkers on the COVID-19 frontline.

## Conclusions

Throughout the COVID-19 crisis, considerable attention has been devoted to ensuring the emotional wellbeing of the country’s senior citizens, particularly during the lockdown period. Untold articles appeared in the Israeli press urging people to be vigilant for symptoms of anxiety and emotional distress among their elderly relatives. Warnings were also published about the possibility that persons being cared for by migrant careworkers unsupervised by family members, may be at increased risk for violence or abuse perpetrated by their careworker [53]. Yet, despite the heightened concern for society’s vulnerable members, we did not encounter a single article in the press addressing the emotional needs of migrant careworkers during the ongoing pandemic. We trust that this report, the first study of the emotional wellbeing of migrant careworkers in Israel during the COVID-19 crisis, will make the “transparent yet indispensable” community of foreign careworkers in Israel [54] more visible.

## Supplementary Information

**Additional file 1.**

## Data Availability

Data is available by request to the corresponding author.

## Abbreviations

HSCL-10: Hopkins Symptom Checklist-10
KLO: Kav L’Oved
OR: Odds ratio
95% CI: 95% confidence interval
