# Supplementary material for: A rapid assessment of migrant careworkers’ psychosocial status during Israel’s COVID-19 lockdown
Source: Isr J Health Policy Res. 2020 Nov 2;9:61. doi: 10.1186/s13584-020-00422-0 (PMC7605873; doi:10.1186/s13584-020-00422-0)
Supplement: Supplementary file 1 — Additional file 1. [file 13584_2020_422_MOESM1_ESM.docx]

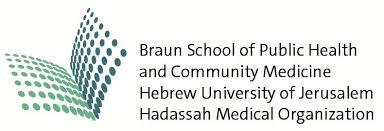

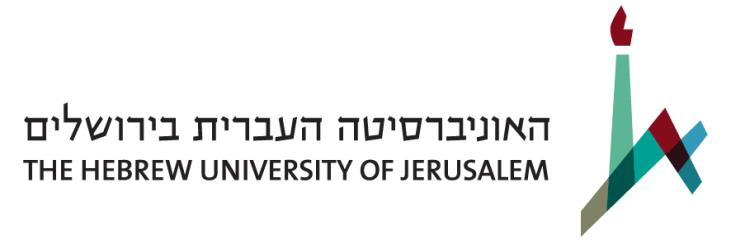


**Questionnaire for Caregivers in Israel during the Covid-19 Pandemic**

**(Distributed online)**

1. Sex
   1. Male
   2. Female
   3. Prefer not to say
2. Age :________________
3. Country of Birth (Nationality):_______________
4. Do you have children?
5. How long have you been in Israel?
   1. Less than 6 months
   2. 6-12 months
   3. 12-62 months
   4. More than 63 months
6. Which visa area do you live in?
   1. Area 1
   2. Area 2
   3. Area 3
7. Do you live with your employer?
   1. Yes, full time
   2. Yes, but I have an apartment for my day off
   3. No
8. How would you describe your physical health in general?
   1. Very good
   2. Good
   3. Average
   4. Poor
   5. Very poor
9. The following are problems people have sometimes. Please mark how often you have felt these things in the last two weeks

|  | Not at all | A little | Quite often | Extremely |
| --- | --- | --- | --- | --- |
| Suddenly feeling scared for no reason |  |  |  |  |
| Feeling fearful |  |  |  |  |
| Faintness, dizziness, or weakness |  |  |  |  |
| Feeling tense or keyed up |  |  |  |  |
| Blaming yourself for things |  |  |  |  |
| Difficulty falling asleep or staying asleep |  |  |  |  |
| Feeling blue (sad or unwell) |  |  |  |  |
| Feeling worthless |  |  |  |  |
| Feeling everything is an effort |  |  |  |  |
| Feeling hopeless about the future |  |  |  |  |

1. Compared to before the outbreak, is your employer’s family visiting:
   1. More often
   2. Less often
   3. About the same
2. How do you access changes to the Israeli government’s Covid-19 guidelines?
   1. Israeli news in English
   2. Israeli news in Hebrew
   3. Israeli news in Russian
   4. My employer explains the guidelines to me
   5. My employer’s family explains the guidelines to me
   6. Other caregivers
   7. My manpower agency
   8. Other organizations (Kav L’Oved, HaKeren)
   9. I don’t hear about guideline changes
3. How confident do you feel to protect yourself and your employer during the Covid-19 outbreak:
   1. Very confident
   2. Confident
   3. Somewhat Confident
   4. A little Confident
   5. Not at all confident
4. Since the start of the Covid-19 outbreak, do you have access to:

|  | Always | Sometimes | Never |
| --- | --- | --- | --- |
| Soap and water |  |  |  |
| Hand Sanitizer (60% alcohol or higher) |  |  |  |
| Surgical Masks |  |  |  |
| N95 Masks |  |  |  |
| Nitrile gloves |  |  |  |
| Laundry machine to wash your clothes |  |  |  |
| Contact with employer’s doctor |  |  |  |
| Food for your employer |  |  |  |
| Food for yourself |  |  |  |

1. In your daily life, have any of the following happened to you since the beginning of the Covid-19 outbreak?

|  | Yes | No |
| --- | --- | --- |
| You have been called names or insulted |  |  |
| You have been threatened or harassed |  |  |
| People act as if they are afraid of you |  |  |
| People have covered their faces while walking past you |  |  |
| People have accused you of having Corona |  |  |
